# Quantifying the unique needs of women with lower limb loss: A national, cross-sectional, survey-based needs assessment

**DOI:** 10.1371/journal.pone.0359172

**Published:** 2026-10-12

**Authors:** Alexis N. Sidiropoulos, Roxanne Disla, Alison L. Pruziner, Tamara Bushnik, Timothy E. Moore, Leif M. Nelson, Heidi Klingbeil, Jason T. Maikos

**Affiliations:** 1 Prosthetics and Sensory Aids Service, Veterans Affairs New York Harbor Healthcare System, New York, New York, United States of America; 2 Physical Medicine and Rehabilitation, James J. Peters Veterans Affairs Medical Center, New York, New York, United States of America; 3 National Veterans Sports Programs and Special Events, Department of Veterans Affairs, Washington, District of Columbia, United States of America; 4 Extremity Trauma and Amputation Center of Excellence, Falls Church, Virginia, United States of America; 5 Department of Rehabilitation, Walter Reed National Military Medical Center, Bethesda, Maryland, United States of America; 6 Department of Rehabilitation Medicine, Rusk Rehabilitation, New York University Langone Health, New York, New York, United States of America; 7 Statistical Consulting Services, Center for Open Research Resources and Equipment, University of Connecticut, Storrs, Connecticut, United States of America; St Mary's University, UNITED STATES OF AMERICA

## Abstract

**Introduction:**

Despite the increasing population of women with limb loss, data to sufficiently characterize potential gender-based differences are lacking, as scientific literature on this topic is often not specifically designed to assess the unique needs of women. Fragmentary information has indicated that women experience a variety of physical and psychological challenges that differ from those experienced by men, but this information has yet to be fully explored. This is the first exploratory needs assessment to combine population-specific physical and psychosocial assessments, as well as gap items that current measures do not capture.

**Methods:**

In this national, cross-sectional, survey-based needs assessment, individuals with lower limb loss recruited across the Department of Veterans Affairs, Department of Defense, and civilian settings completed an online survey. Responses to the survey generated a subset of quantitative scores across several physical and psychosocial domains to compare outcomes between women and men with lower limb loss. Ordered beta regression and multiple linear regression models were used to test the association of gender with scale outcomes, while controlling for additional covariates.

**Results:**

A total of 229 individuals (130 women, 99 men) completed the needs assessment. Results highlighted decreased levels of aesthetic and functional satisfaction, body image, and social adjustment, as well as higher levels of anxiety, in women with limb loss compared with men. There were no differences in depression, activity restriction, or healthcare needs between women and men.

**Conclusions:**

An important contribution of this exploratory needs assessment was its ability to identify candidate constructs that may serve as building blocks for developing a clinically relevant patient-reported outcome measure for women with lower limb loss. Future research should build on these findings to develop and validate women-specific outcome measures, ultimately supporting limb loss care teams in providing optimal, individualized care for this underserved population.

## Introduction

Women with limb loss are a fast-growing demographic, specifically in the Department of Veterans Affairs (VA) [1]. As part of the Global Burden of Disease Study, the incidence of lower limb loss proximal to the toes in the United States for women outpaced men in 2017 (12.8 vs. 12.4 per 100,000), further indicating the rapid growth of this population [2]. Despite this increase, data to sufficiently characterize potential gender-based differences are lacking [1]. While this demographic has been prioritized as a national focus [1], scientific literature on gender-based limb loss needs has often been narrow in scope or not designed to assess the unique needs of women. Specifically, gender is often used as a covariate in studies focused on other requirements of the general limb loss population, creating a fragmented knowledge base of the distinct needs of women with limb loss, leading to a critical gap in knowledge.

To date, this fragmentary information has indicated that women experience a variety of physical and psychological challenges that differ from those experienced by men [3,4], but this information has yet to be fully explored. The fact that advances in prosthetic devices for individuals with lower limb loss are optimized to meet the needs of men, not women [5], has contributed to women reporting greater dissatisfaction with their prostheses [6]. When age, level of limb loss, and comorbidities are controlled for, men are more likely to experience successful limb fitting than women [4]. Women with lower limb loss also suffer from poorer physical functioning and worse health-related quality of life compared with men [7,8]. Disparities between men and women with lower limb loss also span a variety of other factors including body image, intimacy, and mental health [9–11]. Previous literature has also suggested that gender is an independent factor in the success of rehabilitation post-limb loss, with women experiencing greater difficulties than men, regardless of factors such as age, level of limb loss, and comorbidity [4]. Gender-influenced outcomes also include limb fitting, physical activity, and pain [12,13].

The published literature specific to women with lower limb loss typically includes small sample sizes, tends to be qualitative in nature, and is limited in focus, predominantly addressing psychological and psychosocial factors while largely omitting prosthetic outcomes and functional performance data stratified by gender [14–16]. Unfortunately, no data to sufficiently characterize potential gender differences correlated with the prosthetic-based needs of this subpopulation exist. This population’s needs must be met by the evolution of both healthcare delivery and research practices by successfully developing evidence-based interventions to evaluate all domains related to women’s health. This study is the first exploratory needs assessment to combine population-specific physical and psychosocial assessments, as well as gap items developed by a multidisciplinary expert panel to capture domains not addressed by existing measures, including interactions with healthcare providers, social support, sexual health, employment, and role in the residence (Table 1) [17]. Therefore, the purpose of the investigation was to assess the unique impact of lower limb loss on women compared with men, including physical, functional, psychosocial, and healthcare needs, with the long-term goal of informing the development of women-specific outcome measures. Due to the previous fragmented research specific to the needs of women with limb loss, we proposed a general hypothesis that women with lower limb loss would have distinct physical, functional, psychosocial, and healthcare needs compared with men with lower limb loss.

**Table 1:** Standardized questionnaires and non-standardized supplemental gap item domains included in the online needs assessment survey for individuals with limb loss.

**Non-Limb Loss-Specific Surveys**	PROMIS Global Health
	PROMIS Emotional Support 8a
	PROMIS Depression 8a
	PROMIS Anxiety 8a
	PROMIS Pain Interference 8a
**Limb Loss-Specific Surveys**	Trinity Amputation and Prosthesis Experience Scales – Revised
	Orthotics and Prosthetics Users’ Survey: Satisfaction with Services
	Prosthesis Evaluation Questionnaire Domains: Appearance Scale, Residual Limb Health Scale
	Prosthesis Evaluation Questionnaire Questions: Pain, Transfers, Importance
	Amputee Body Image Scale – Revised
**Gap Item Domains**	Demographics
	Interaction and comfort with healthcare providers
	Effect of limb loss on social support
	Effect of limb loss on sexual health
	Effect of limb loss on employment status
	Effect of limb loss on role in the residence

PROMIS, Patient Reported Outcome Measurement Information System.

## Materials and methods

This study was a national, cross-sectional, self-reported, survey-based needs assessment to aid in understanding the unique needs of women with lower limb loss [17]. This investigation was a collaboration between the VA, Department of Defense (DoD), and the private sector. Women and men with major lower limb loss completed the survey-based needs assessment anonymously and electronically without individually identifiable information through an online database developed in conjunction with the Army Public Health Center. This study was approved by the VA Central Institutional Review Board (#18–20) and oversight was provided by the U.S. Army Medical Research and Development Command Office of Human Research (log number E04083). This study was reported in accordance with the Strengthening the Reporting of Observational Studies in Epidemiology (STROBE) guidelines for cross-sectional studies (S1 Checklist).

Veterans, service members, and civilians with major lower limb loss (i.e., ankle disarticulation or more proximal) were recruited across the United States to complete the survey-based needs assessment in several ways. First, a review of VA regional amputation centers and polytrauma amputation networks was conducted to identify VA facilities with the largest population of female veterans with lower limb loss. Providers at 10 of these VA sites agreed to recruit eligible male and female veterans with major lower limb loss from their local prosthetics and physical medicine and rehabilitation clinics.

Additionally, veterans with major lower limb loss were recruited at the 2019 VA National Disabled Veterans Winter Sports Clinic and the 2019 VA National Veterans Wheelchair Games. Service members with lower limb loss within the DoD were primarily recruited from the Department of Rehabilitation at Walter Reed National Military Medical Center. Civilians with lower limb loss were recruited from the 2019 Amputee Coalition National Conference and the 2019 Amputee Coalition Limb Loss Education Day.

Lastly, due to the online nature of the survey-based needs assessment, supplemental recruitment strategies included postings on VA and DoD social media outlets and listservs, presentations at local and virtual limb loss support groups, and postings on patient organization websites. Collectively, these recruitment methods ensured a large and diverse sample. Eligible participants were at least 18 years old, had been living with major lower limb loss for at least one year, and were able to effectively read or write in English. Recruitment and data collection occurred from July 2, 2019 to July 2, 2022.

Several strategies were employed to minimize potential sources of bias. To reduce selection bias, a diverse, multi-sector recruitment approach was used across VA, DoD, and civilian settings nationwide. To minimize response bias, the survey was completed anonymously and electronically without individually identifiable information. To decrease bias resulting from a technology gap, participants were provided free internet access and hardware on site. Use of standardized, validated instruments across all domains further reduced the potential for measurement bias.

The development and pilot testing of the survey-based needs assessment have been previously described in detail [17]. In brief, this assessment included screening and demographic questions, valid and reliable limb loss and non-limb loss-specific surveys, and supplemental gap questions written by the investigative team and iteratively refined with a multidisciplinary expert panel. The content (Table 1) was arranged around domains for general health, quality of life, prosthetic use and needs, psychosocial health and behaviors, and body image. After initial consent to participate, the online survey asked a series of screening and demographic questions to determine eligibility.

Demographic questions included items on gender, age, weight, height, race/ethnicity, marital status, employment, education, living situation (e.g., alone/spouse/roommates, family, assistive care), military status, and health diagnoses or conditions. Gender was self-reported; therefore, gender refers to self-identified gender for both men and women. Limb loss screening/demographic questions included level of limb loss, time since limb loss, etiology of limb loss, and history of prosthesis use. Participants were exited from the survey if they reported that they did not have major lower limb loss or had major lower limb loss for less than one year. All eligible participants then answered the same bank of survey questions. Individuals with bilateral lower limb loss completed the same limb loss-specific questions for each side. The survey-based needs assessment was designed to be completed in a single session of 30–45 minutes.

### Statistical analysis

Data cleaning was performed using SPSS (IBM Statistics, Armonk, NY) [18] and all statistical analyses were conducted in R (version 4.3.3) [19]. The primary goal of the analyses was to compare outcomes between women and men with lower limb loss. Descriptive statistics were generated for all demographic variables. Continuous variables, including height and weight, were summarized as mean and standard deviation. Categorical variables, including age, level of limb loss, time since limb loss, etiology, ethnicity, race, military status, education, employment status, relationship status, and residence, were summarized as counts. Differences in demographic characteristics between women and men were assessed using chi-square proportion tests, with statistical significance set at α = 0.05. Regression models were used to test the association of gender with scale outcomes, while adjusting for additional covariates selected *a priori* based on theoretical relevance to the outcomes of interest. Specifically, age, PROMIS Global Mental and Physical Health scores, etiology, and time since limb loss were included as covariates, as each represents a factor with established potential to influence physical and psychosocial outcomes in the limb loss population [20–23]. PROMIS Global Mental and Physical Health scores were included to capture overall self-reported health status that could systematically influence survey responses independent of gender. For bounded outcomes (i.e., data with upper and lower limits), ordered beta regression models, a recently developed approach for handling bounded data were used [24]. These ordered beta regression models were fit in a Bayesian framework, using the ordbetareg package [24]. Statistical significance was determined by whether the 95% credible interval included zero.

For PROMIS T-score outcomes, linear regression models were used. Significance for linear regression models was determined by whether the 95% confidence interval excluded zero, consistent with a two-sided α = 0.05 threshold. Confidence intervals were reported to quantify the magnitude and precision of effects, allowing for assessment of both statistical and practical importance of the results.

S1 Table details the statistical model used for each outcome measure. Both models were checked for model fit, including normality of residuals (i.e., linear regression models), outliers, homoscedasticity, and posterior predictions using the performance package [25]. No subgroup analyses or interaction terms were examined. Participants with missing data for a given outcome were excluded from the corresponding analysis, resulting in varying sample sizes across outcomes, which are reported in S1 Table. All analyses reflect a convenience sample; therefore, findings should be interpreted in the context of a non-probability sampling design. Sample size justification and a priori power analysis have been described in detail in the previously published study protocol [17]. Briefly, a total sample size of 192 participants was required to achieve 80% power, and a convenience sample of 200 individuals (100 per group) was proposed.

## Results

S1 Table details the results for all survey outcomes. The subsequent sections detail selected demographics and outcomes for physical, functional, healthcare, and psychosocial needs. Demographic characteristics of study participants are presented in Table 2. Of the 431 individuals who accessed and were screened for the survey, 57 were excluded for not meeting eligibility criteria. Of the 374 individuals who met eligibility criteria and proceeded to the full survey, 229 completed the needs assessment (130 women, 99 men). Reasons for non-completion were not collected; however, no survey responses were mandatory except for inclusion criteria questions (i.e., at least one year since limb loss, age, and gender), and all responses provided were included in analyses. S1 Table provides the number of responses per survey question for women and men separately. Response counts (Table 2) for certain variables may exceed the reported sample size for two reasons: bilateral participants reported level of limb loss and time since limb loss for each affected limb, and participants were permitted to select all applicable responses for variables including etiology of limb loss and race. Height and weight had notably low response rates (23% and 28%, respectively), precluding the ability to control for BMI in the statistical analyses; however, participants with missing height and weight data were retained in all subsequent analyses, preserving statistical power and representativeness of the findings. Significantly more women completed the needs-based survey than men, and women indicated higher levels of education compared with men. Men were observed to be taller and heavier than women. Most limb loss incidences were a result of infection, and significantly more civilians completed the survey compared with veterans and active service members.

**Table 2:** Demographics.

	Women (130)	Men (99)	Overall (229)
**Age**			
< 35 *	39	13	52
35-64	70	60	130
> 64	21	26	47
**Height** (in)			
Mean (SD) *	64.2 (4.2)	70.6 (3.1)	66.9 (4.9)
**Weight** (lbs)			
Mean (SD) *	160.5 (47.5)	200.0 (52.7)	179.0 (53.7)
**Level of Limb Loss**			
Ankle Disarticulation	2	2	4
Transtibial ^*^	68	72	140
Knee Disarticulation	4	3	7
Transfemoral ^*^	66	33	99
Hip Disarticulation	7	3	10
Hemipelvectomy	1	0	1
**Etiology of Limb Loss**			
Infection	37	35	72
Car or motorcycle accident	33	25	58
Other Trauma	16	10	26
Cancer	15	9	24
Diabetes	6	16	22
Vascular Disease	11	6	17
Since Birth ^*^	16	1	17
Other ^*^	13	3	16
Explosive Device ^*^	1	5	6
Gunshot	2	1	3
Prefer not to answer	1	2	3
**Time Since Limb Loss**			
1–2 years	29	16	45
2–5 years	41	22	63
5–10 years	21	22	43
> 10 years	53	50	103
**Ethnicity**			
Not Hispanic or Latino	119	86	205
Hispanic or Latino	10	12	22
Not Reported	1	1	2
**Race**			
White	111	77	188
Black or African American	12	16	28
Other	6	5	11
American Indian or Native Alaskan	1	1	2
Asian ^*^	5	0	5
Not Reported	2	2	4
**Military Status**			
Civilian ^*^	107	54	161
Veteran ^*^	22	43	65
Active Service Member	0	1	1
Other	1	1	2
**Education Level**			
Some High School	3	1	4
High School Graduate ^*^	7	15	22
Trade or Vocation School Cert ^*^	2	11	13
Some College	22	24	46
Associate’s Degree	16	16	32
Bachelor’s Degree ^*^	51	19	70
Master’s Degree ^*^	23	6	29
Doctorate or Other Prof Degree	6	6	12
Unknown	0	1	1
**Employment Status**			
Employed	62	39	101
Retired ^*^	36	43	79
Unemployed	20	10	30
Other	12	7	19
**Relationship Status**			
Married	55	51	106
Divorced	27	27	54
Never Married	36	16	52
Widowed	7	3	10
Other	4	1	5
Separated	1	0	1
Unknown	0	1	1
**Residence**			
With Sig Other	60	53	113
Private Residence	30	24	54
With Family	24	15	39
With Roommates – not significant other	8	2	10
Other	6	1	7
Shelter	1	2	3
Assisted Care Facility	0	1	1
Prefer not to answer	1	1	2

*Significantly different between women and men based on X^2^ proportion test (α = 0.05); Response counts for level of limb loss and time since limb loss may exceed total sample size due to bilateral participants reporting data for each affected limb. Response counts for etiology of limb loss and race may exceed total sample size as participants were permitted to select all applicable responses.

### Physical needs

Women scored lower in satisfaction with appearance (4.7 ± 1.2 vs. 5.7 ± 1.1), importance for maintaining limb health (4.2 ± 1.2 vs. 4.8 ± 1.1), and aesthetic satisfaction (6.5 ± 1.9 vs. 7.5 ± 1.6), though the residual limb health score was not different from men (4.6 ± 1.5 vs. 5.2 ± 1.2). See S1 Table for complete descriptive statistics. The distribution of physical needs outcomes for women and men is presented in Fig 1.

**Fig 1:**
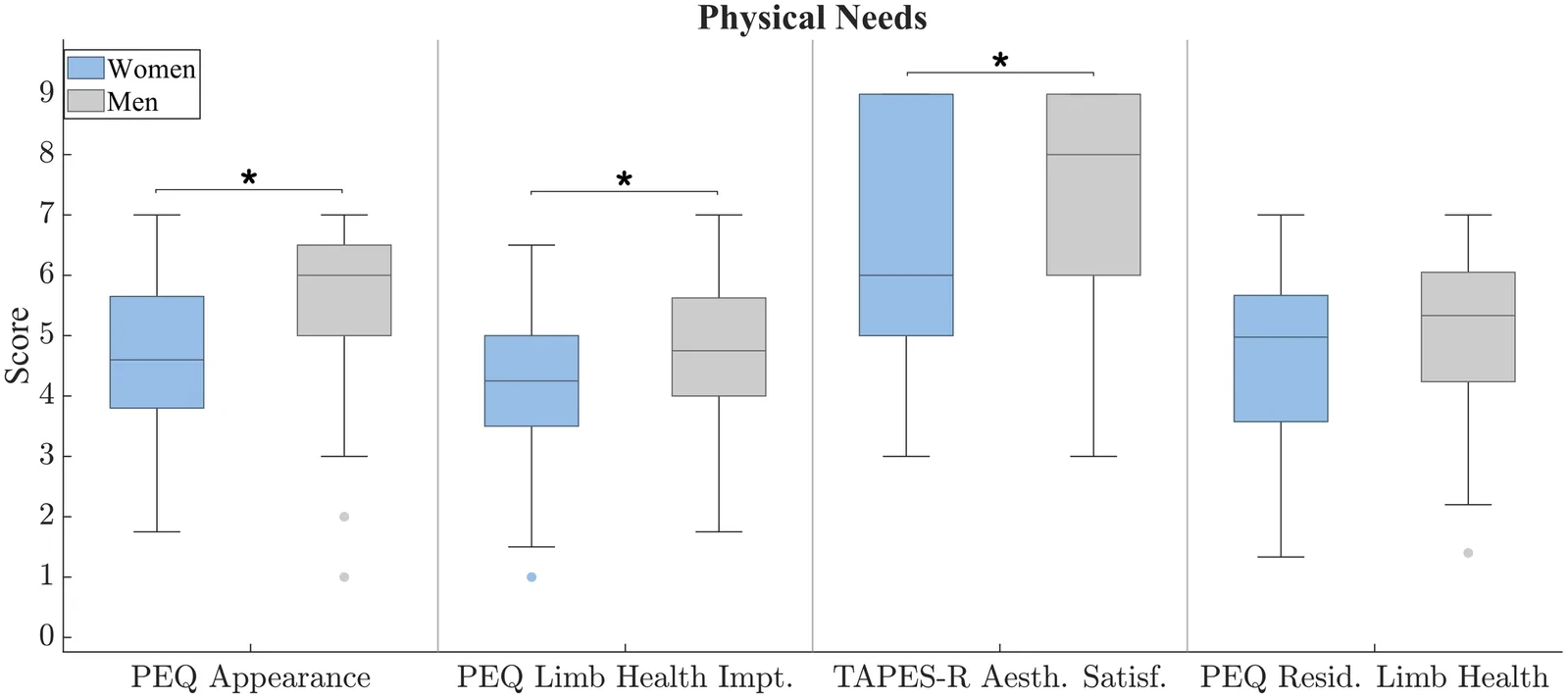
Difference in physical needs between women and men with lower limb loss. PEQ: Prosthesis Evaluation Questionnaire, Impt: Importance, TAPES-R: Trinity Amputation and Prosthesis Experience Scales – Revised, Aesth: Aesthetic, Resid: Residual, Satisf: Satisfaction. * denotes outcomes where the 95% credible interval from the ordered beta regression did not include zero, indicating a significant difference between women and men.

### Functional needs

Women indicated lower functional satisfaction than men (10.3 ± 3.1 vs. 11.7 ± 2.8), but no differences in activity restriction were observed between women and men (1.4 ± 0.3 vs. 1.5 ± 0.4). See S1 Table for complete descriptive statistics. The distribution of functional needs outcomes for women and men is presented in Fig 2.

**Fig 2:**
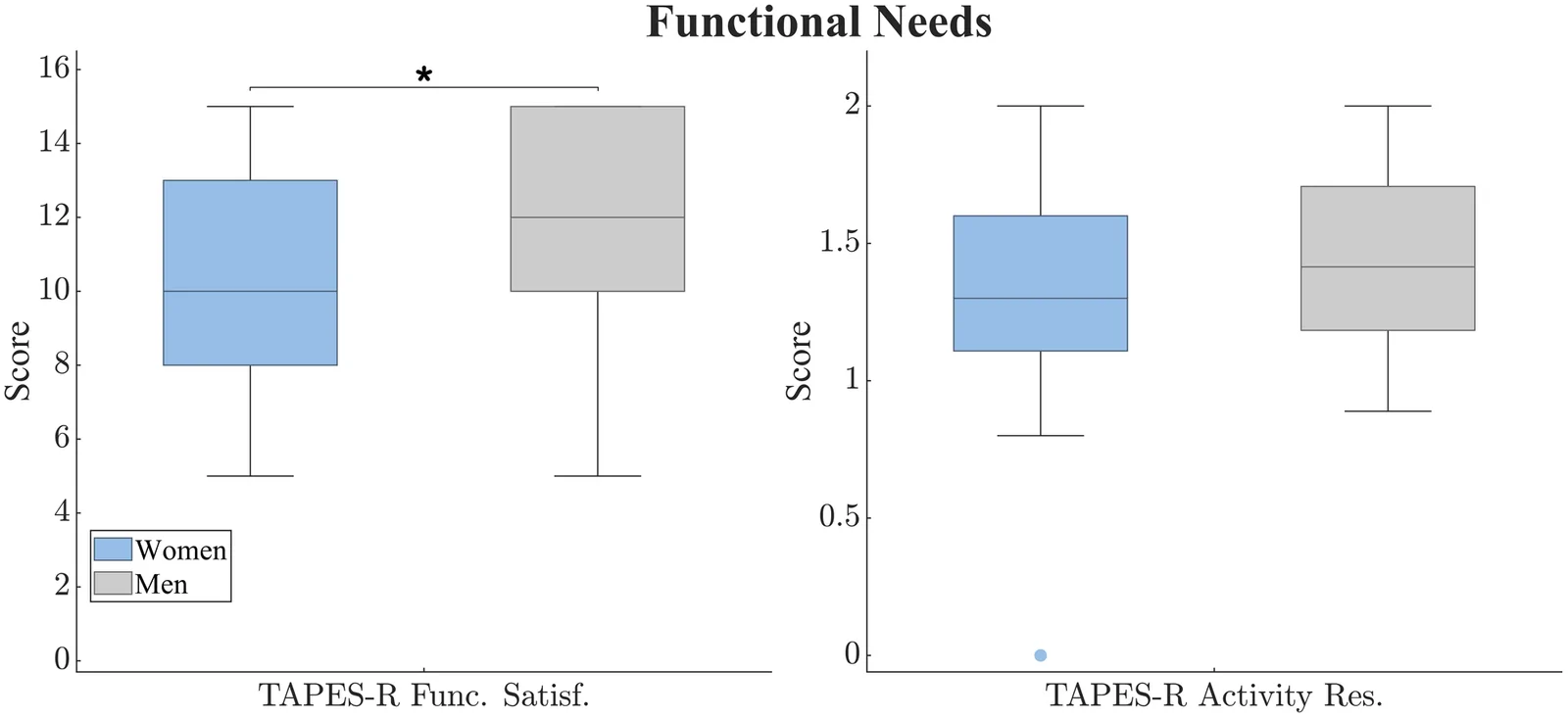
Difference in functional needs between women and men with lower limb loss. TAPES-R: Trinity Amputation and Prosthesis Experience Scales – Revised, Func: Functional, Satisf: Satisfaction, Res: Restriction. * denotes outcomes where the 95% credible interval from the ordered beta regression did not include zero, indicating a significant difference between women and men.

### Healthcare needs

No differences between women and men were observed regarding healthcare needs. Specifically, there were no differences in satisfaction with services (7.9 ± 4.3 vs. 7.5 ± 3.8) or comfort with their primary care provider (6.0 ± 1.8 vs. 6.0 ± 1.8), prosthetist (6.1 ± 1.6 vs. 6.1 ± 1.8), or other providers (6.1 ± 1.7 vs. 6.1 ± 1.9). See S1 Table for complete descriptive statistics. The distribution of healthcare needs outcomes for women and men is presented in Fig 3.

**Fig 3:**
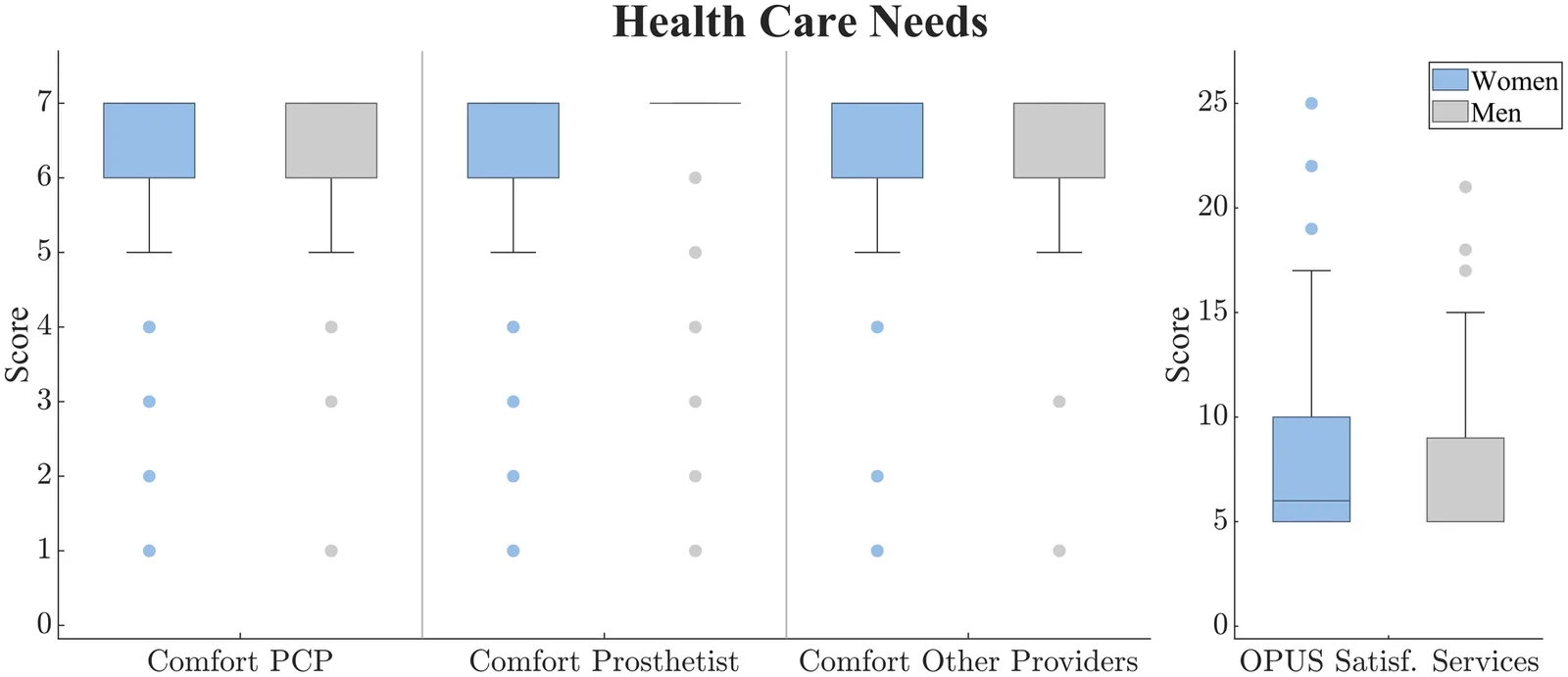
Difference in healthcare needs between women and men with lower limb loss. PCP: Primary Care Provider, OPUS: Orthotics and Prosthetics Users’ Survey, Satisf: Satisfaction.

### Psychosocial needs

Women had lower social adjustment (2.9 ± 0.5 vs. 3.1 ± 0.3) than men. Women also indicated less comfort in social situations with people they are sexually attracted to (4.1 ± 2.1 vs. 5.2 ± 2.0), while reporting higher levels of comfort in intimate situations with friends (5.3 ± 1.7 vs. 4.8 ± 1.8) and family members (5.3 ± 1.9 vs. 5.0 ± 1.9) compared with men. Women indicated higher body image concern (9.5 ± 5.3 vs. 7.1 ± 4.7) and higher levels of anxiety (53.6 ± 10.1 vs. 49.4 ± 10.3) compared with men. There were no differences in levels of depression (52.0 ± 10.8 vs. 48.7 ± 10.2), pain interference (56.8 ± 9.5 vs. 55.3 ± 8.9), or emotional support (51.4 ± 9.1 vs. 53.4 ± 9.4) between women and men. See S1 Table for complete descriptive statistics. The distribution of psychosocial needs outcomes for women and men is presented in Fig 4.

**Fig 4:**
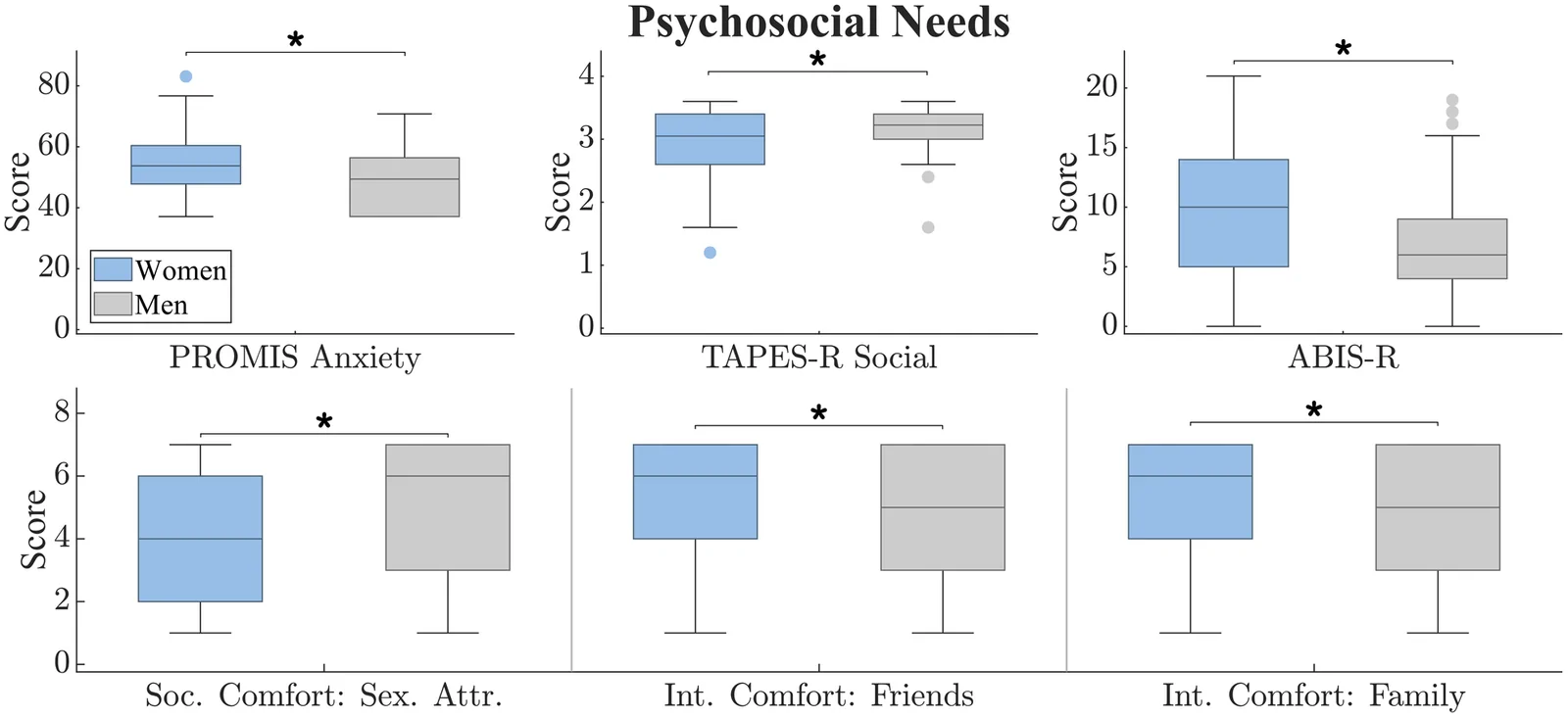
Difference in psychosocial needs between women and men with lower limb loss. Soc: Social, Sex: Sexually, Attr: Attracted, Int: Intimate, TAPES-R: Trinity Amputation and Prosthesis Experience Scales – Revised, ABIS-R: Amputee Body Image Scale – Revised. * denotes outcomes where the 95% credible interval (ordered beta regression) or 95% confidence interval (linear regression) did not include zero, indicating a significant difference between women and men.

## Discussion

There is a rapidly growing population of women with limb loss, yet fragmented research has been conducted to understand the unique needs of this population [1,26]; however, it is critical to identify both the physical and psychosocial needs of women with limb loss to provide evidentiary support for clinical decision making in this demographic. This study constitutes the first exploratory needs assessment aimed at examining the multidimensional challenges faced by this population. Given the cross-sectional design, findings should be interpreted as preliminary and hypothesis-generating rather than definitive characterizations of gender-based needs. Supporting our hypothesis, results identified differences between women and men with lower limb loss. A significant benefit of this needs assessment was its ability to generate a subset of quantitative scores across several physical and psychosocial domains, which can be useful for identifying differences in physical and psychosocial needs in this population based on situational factors and salient constructs related to health and wellbeing of women with lower limb loss.

### Physical needs

In agreement with previous literature [7,27], women with lower limb loss in this study indicated lower scores associated with appearance and aesthetic satisfaction compared with men with lower limb loss. Appearance of a prosthetic device affects device acceptance, and it has been noted that cosmetic appearance can play as great a role in the psychological aftermath of an amputation as the return of physical function [28]. While greater aesthetic quality of prosthetic devices may improve body image and psychological well-being in women with lower limb loss [29], most of the over 350 commercially available prosthetic ankle-foot devices [30] have been designed to meet male anthropometry and biomechanics.

Consequently, women have fewer prosthetic options, specifically for ankle-foot devices [31], due to their smaller stature compared with men [31], which requires smaller prosthetic components. Though manufacturers have recently started to create women-specific prosthetic devices [31], none are advertised well or easily identified on their respective websites. Some devices are limited in size and stiffness and are not rated for higher impact activities, reducing their applicability to the general population of women with lower limb loss. For these reasons, limb loss care providers often prescribe pediatric ankle-foot devices to meet the required size and weight categories [32]. These devices are not designed to meet the rigorous biomechanical needs of an adult population, potentially leading to ill-fitting prescriptions and physical consequences such as increased skin integrity problems and secondary comorbidities [33]. While no significant differences in residual limb health were observed between women and men in this study, the literature specifically examining sex and gender differences in residual limb health remains limited. One study found that male sex was a protective determinant for residual limb skin problems in a large cohort of lower limb prosthesis users [34], suggesting this may be an area warranting further investigation, particularly given the documented challenges women face in obtaining well-fitting prosthetic devices. Collectively, these factors often result in higher rates of artificial limb rejection for women with lower limb loss [35]. Therefore, these findings suggest that limb loss care providers should consider the unique needs of their female patients when addressing deficiencies in commercially available devices, with the goal of elevating the standard of care for this underserved population.

### Functional needs

Women with lower limb loss reported lower functional satisfaction compared with men in this study, which can often lead to reduced participation in activities. However, both men and women reported similar, high levels of activity restriction (see S1 Table), which is consistent with previous literature [36]. Activity restriction and functional dissatisfaction for individuals with lower limb loss are major concerns due to the known deleterious effects of a sedentary lifestyle [37,38]. While the positive physical and psychosocial effects of physical activity are well established, individuals with disabilities participate less than the general population due to complications of both internal (e.g., motivation, confidence, competence, enjoyment) and external/environmental factors (e.g., access to facilities/equipment, social support, time constraints, costs, recognition) [39].

### Healthcare needs

Interestingly, the results from this study indicated no discrepancy between women and men in satisfaction with prosthetic services or level of comfort with their limb loss care providers. This contrasts with previous qualitative research in which lack of individualized care and the need for better access to female prosthetists were common themes for women with limb loss [5]. This may be due to the open-ended nature of qualitative research that allows for greater expression compared with quantitative research which asks more pointed questions that do not permit expansion of a response. These divergent results are not entirely surprising since each measurement technique was based on fundamentally different theoretical paradigms and sample characteristics, even though both sets of questions were related to satisfaction with limb loss care. These outcomes should be treated as complementary, each underpinning the complexity inherent in understanding the unique needs of this population, indicating the need for further research.

### Psychosocial needs

In this study, women reported higher levels of anxiety than men, though scores for both groups were within one standard deviation of the U.S. general population [40–42]. There were no differences between women and men for depression, pain interference, or emotional support. It should be noted that PROMIS measures of depression, anxiety, pain interference, and emotional support were used as outcome measures in this study. While these measures are well-validated in the general adult population, they have not been specifically validated for individuals with limb loss. This distinction is relevant to construct validity, as items within these measures may not fully capture the unique ways in which limb loss influences social functioning and mood. As such, findings from these measures should be interpreted with caution, as responses may reflect broader life experiences rather than limb loss-specific phenomena. Previous research examining psychosocial adaptations to limb loss, most commonly captured as prevalence of depression and anxiety, has reported mixed results. For example, some studies have found that individuals with lower limb loss are prone to higher levels of depression, with a prevalence as high as 46% [43], whereas other studies have found no evidence of increased levels of depression [12]. Psychological symptoms tend to vary considerably at different stages over the lifetime of individuals with limb loss, with the highest levels reported during the acute phase of limb loss (up to two years), specifically for depression [44] and anxiety [45]. Because most psychosocial analyses in limb loss, including this study, have been cross-sectional in design, these conflicting results are likely due to the complexities of measuring fluid psychological outcomes at discrete time points. The finding of higher anxiety in women with lower limb loss aligns with broader literature demonstrating that women in the general population report higher levels of anxiety than men [46–48]. Whether the observed difference in this sample reflects a limb loss-specific phenomenon or a continuation of population-level sex differences in anxiety cannot be determined from the present cross-sectional data.

Additionally, the dichotomy in the scientific literature may be shaped by the heterogeneity of different participant characteristics (e.g., age, etiology, level of limb loss, time since limb loss, etc.) and different data collection methods and tools [49]. Previous studies have also shown mixed results in psychosocial outcomes between men and women with limb loss. Some studies reported gender-based differences [50] while others reported no differences [44], likely due to heterogeneity of sample characteristics and underrepresentation of women. Given the potential for psychosocial distress at various points across the lifespan in this population, and the potential impact of anxiety on rehabilitation engagement, coping with prosthesis use, and overall quality of life, the interdisciplinary limb loss care team should routinely monitor these outcomes to limit the adverse effects of elevated psychosocial stressors on general health outcomes [51] and provide effective coping strategies when appropriate [52].

### Body image

Evaluation of body image has been of particular interest in the limb loss population due to its association with general adjustment to limb loss [53], psychosocial parameters, and identity [54]. Body image for individuals with lower limb loss has been extensively studied across levels of limb loss [55], as well as compared with subgroups [56–61]. Most studies indicated that individuals with lower limb loss had worse body image perception compared with many subgroups [56,57], and reported their body as being incomplete or worth less following limb loss [62]. Of the few studies that have evaluated body image between women and men with limb loss, conflicting results were reported as one study indicated that women had significantly better perceived body image compared with men at three months post-limb loss [14], whereas another study reported no differences for individuals with chronic limb loss [15].

Results from this study indicated that women with lower limb loss had worse body image compared with men. For this population, body image can also play a significant role in comfort level in specific situations, as previous literature has indicated that the negative body image experienced post-limb loss has correlated to the struggle women encounter with reconciling their post-limb loss representations of themselves to new ways of functioning [62]. This discomfort may lead to an overall lower and more difficult adjustment to new situations compared with men. Given the lack of research comparing body image after limb loss in men and women, specifically throughout the lifetime of the person, expanded research will be critical to understand the magnitude of body image perception to develop effective interventions to improve quality of life.

In this study, women exhibited lower levels of social adjustment to their limb loss compared with men. To further explore post-limb loss social adjustment, supplemental questions surrounding comfort in social, intimate, and sexual situations were included in this needs assessment [17]. Women reported lower levels of comfort in social situations with people they were sexually attracted to compared with men, while no other differences for comfort in sexual situations were found. Interestingly, women reported higher levels of comfort in intimate situations with friends and family members compared with men, indicating a level of trust with familiar members of their social circle post-limb loss.

## Conclusion

This study represented the first comprehensive, survey-based needs assessment to aid in understanding the unique needs of women with lower limb loss, providing a foundation for the future development of women-specific outcome measures. Study results highlighted the decreased levels of aesthetic and functional satisfaction, body image, and social adjustment, as well as higher levels of anxiety in women compared with men. There were no differences in depression, activity restriction, or healthcare needs between women and men. While appropriate healthcare for individuals with lower limb loss stems from evidence-based research, there is a disparity between male and female research participants in lower limb loss research as women are studied significantly less than men, specifically in high validity study types such as randomized control trials and systematic reviews [63]. Studies that predominantly used male participants without a commensurate level of female representation to develop “gold-standard” devices, techniques, and tools in lower limb loss may be inherently flawed for treating or serving the needs of women [63]. As such, use of these tools by women with lower limb loss may result in suboptimal outcomes, perpetuating a negative feedback cycle of recurring challenges. An important contribution of this exploratory, survey-based needs assessment was its ability to identify candidate constructs that may serve as building blocks for developing a clinically relevant patient-reported outcome measure for women with lower limb loss. While existing outcome measures are sufficient to identify broad differences between women and men with lower limb loss, they may lack the sensitivity to detect meaningful change in women-specific constructs following targeted interventions. Women-specific outcome measures would therefore serve a dual purpose: providing justification for changes to clinical care and product development, and enabling more precise tracking of progress in response to women-specific interventions. Future research should build on these findings to develop and validate women-specific outcome measures, ultimately supporting limb loss care teams in providing optimal, individualized care for this underserved population.

### Limitations

Several limitations should be considered when interpreting these findings. As a self-reported survey study, reporting, recall, and volunteer bias may have influenced participant responses; however, the diverse, multi-sector recruitment strategy, including both clinic-based and community recruitment, may have partially mitigated volunteer bias. As the survey was distributed through multiple channels, non-response bias could not be assessed. Available-case analysis was used since survey responses were not mandatory, though the overall pattern of findings is unlikely to be substantially affected given the large and diverse sample. Given the large number of outcomes examined across multiple domains without adjustment for multiple comparisons, the possibility of Type I error cannot be excluded, and significant findings should be interpreted with caution. However, use of Bayesian ordered beta regression models provides stable inference for bounded, non-normal outcomes and reduces the likelihood of false positive findings. Conversely, for outcomes where no gender differences were observed, the possibility of Type II error cannot be excluded, and these null findings should not be interpreted as evidence of equivalence. All findings should be considered hypothesis-generating and require replication in future confirmatory studies. Although key demographic variables were included as covariates in the regression models, residual confounding cannot be excluded, as observed differences between women and men in age distribution, military status, and education level may have influenced outcomes in ways not fully captured by the selected covariates. Due to the lack of racial and ethnic diversity and the English-only survey requirement, this study may not be as generalizable to Hispanic/Latino, non-Caucasian, or non-English-speaking individuals with limb loss. Additionally, the predominance of civilian participants may limit generalizability to the VA and DoD populations specifically. The national recruitment scope, however, strengthens the generalizability of findings to the broader U.S. population of individuals with major lower limb loss. While infection was the leading etiology of limb loss, underlying conditions contributing to infection may not have been fully captured. Some outcome measures (i.e., PROMIS measures) have not been specifically validated for individuals with limb loss, which has implications for construct validity, as responses may reflect broader life experiences rather than limb loss-specific phenomena. Finally, due to the cross-sectional design, correlational data were presented, but causal inferences cannot be confirmed.

## Supporting information

S1 Table Complete descriptive statistics and statistical model results for all survey outcomes. This table provides descriptive statistics (mean and standard deviation) and full regression model results (estimates and 95% credible or confidence intervals) for all survey outcomes, presented separately for women and men with lower limb loss. Ordered beta regression results (A) are reported for bounded outcomes, and linear regression results (B) are reported for PROMIS T-score outcomes.
(DOCX)

S1 Checklist STROBE Statement checklist for cross-sectional studies. Completed STROBE (Strengthening the Reporting of Observational Studies in Epidemiology) checklist documenting adherence to reporting standards for cross-sectional observational studies.
(DOCX)
