# Supplementary material for: Quantifying the unique needs of women with lower limb loss: A national, cross-sectional, survey-based needs assessment
Source: PLoS One. 2026 Oct 12;21(10):e0359172. doi: 10.1371/journal.pone.0359172 (PMC13657298; doi:10.1371/journal.pone.0359172)
Supplement: Supplementary file 1 [file pone.0359172.s001.docx]

| **S1 Table. Complete descriptive statistics and statistical model results for all survey outcomes.** This table provides descriptive statistics (mean and standard deviation) and full regression model results (estimates and 95% intervals) for all survey outcomes, presented separately for women and men with lower limb loss. Ordered beta regression results (A) are reported for bounded outcomes and linear regression results (B) are reported for PROMIS T-score outcomes. | | | | | |
| --- | --- | --- | --- | --- | --- |
| A. **Ordered Beta Results** | | | | **Ordered Beta** | |
|  | **Parameter** | **Sample Size (n)** | **Mean (Std)** | **Estimates** | **Credible Intervals (95%)** |
| *Physical Needs* | PEQ Appearance | Women (117) | 4.7 (1.2) | **-0.6** | **[-0.8 ̶ -0.4]** |
|  |  | Men (90) | 5.7 (1.1) |  |  |
|  | PEQ Imp for Maint Limb Health | Women (116) | 4.2 (1.2) | **-0.3** | **[-0.5 ̶ -0.1]** |
|  |  | Men (90) | 4.8 (1.1) |  |  |
|  | PEQ Residual Limb Health Scale | Women (117) | 4.6 (1.5) | -0.2 | [-0.5 ̶ 0.1] |
|  |  | Men (89) | 5.2 (1.2) |  |  |
|  | TAPES-R Aesthetic Satisfaction | Women (113) | 6.5 (1.9) | **-0.4** | **[-0.7 ̶ -0.2]** |
|  |  | Men (87) | 7.5 (1.6) |  |  |
| *Functional Needs* | TAPES-R Functional Satisfaction | Women (113) | 10.3 (3.1) | **-0.3** | **[-0.6 ̶ -0.0]** |
|  |  | Men (88) | 11.7 (2.8) |  |  |
|  | TAPES-R Activity Restriction | Women (113) | 1.4 (0.3) | -0.1 | [-0.4 ̶ 0.2] |
|  |  | Men (84) | 1.5 (0.4) |  |  |
| *Healthcare Needs* | OPUS Satisfaction with Services | Women (115) | 7.9 (4.3) | 0.1 | [-0.2 ̶ 0.4] |
|  |  | Men (90) | 7.5 (3.8) |  |  |
|  | Comfort with PCP | Women (119) | 6.0 (1.8) | 0.1 | [-0.2 ̶ 0.6] |
|  |  | Men (89) | 6.0 (1.8) |  |  |
|  | Comfort with Prosthetist | Women (117) | 6.1 (1.6) | 0.1 | [-0.3 ̶ 0.5] |
|  |  | Men (94) | 6.1 (1.8) |  |  |
|  | Comfort with Other Providers | Women (112) | 6.1 (1.7) | -0.1 | [-0.4 ̶ 0.3] |
|  |  | Men (82) | 6.0 (1.9) |  |  |
| *Psychosocial Needs* | ABIS-R Body Image | Women (130) | 9.5 (5.3) | **0.4** | **[0.2 ̶ 0.6]** |
|  |  | Men (99) | 7.1 (4.7) |  |  |
|  | Comfort in Soc Sit with Peers | Women (117) | 5.8 (1.7) | -0.0 | [-0.4 ̶ 0.3] |
|  |  | Men (89) | 6.0 (1.6) |  |  |
|  | Comfort in Soc Sit with people sexually attracted to | Women (105) | 4.1 (2.1) | **-0.4** | **[-0.7 ̶ -0.1]** |
|  |  | Men (85) | 5.2 (2.0) |  |  |
|  | Comfort in intimate situations with Friends (1) | Women (113) | 5.3 (1.7) | **0.5** | **[0.2 ̶ 0.8]** |
|  |  | Men (88) | 4.8 (1.8) |  |  |
|  | Comfort in Intimate situations with family (2) | Women (110) | 5.3 (1.9) | **0.3** | **[0.0 ̶ 0.6]** |
|  |  | Men (85) | 5.0 (1.9) |  |  |
|  | Comfort in Intimate situations with your children (3) | Women (81) | 5.4 (1.7) | 0.1 | [-0.3 ̶ 0.4] |
|  |  | Men (74) | 5.1 (2.0) |  |  |
|  | Comfort in Intimate Situation with Sig Other (4) | Women (80) | 5.6 (1.8) | 0.4 | [-0.0 ̶ 0.7] |
|  |  | Men (77) | 5.4 (1.7) |  |  |
|  | Comfort in engaging in Sexual Situations with Self (1) | Women (81) | 5.3 (1.9) | -0.2 | [-0.6 ̶ 0.1] |
|  |  | Men (70) | 5.5 (1.8) |  |  |
|  | Comfort in engaging in Sexual Situations with New Partner (2) | Women (47) | 3.4 (2.2) | -0.3 | [-0.8 ̶ 0.2] |
|  |  | Men (46) | 4.0 (2.1) |  |  |
|  | Comfort in engaging in Sexual Situations with Familiar Partner (3) | Women (75) | 4.9 (2.0) | -0.0 | [-0.4 ̶ 0.3] |
|  |  | Men (71) | 5.0 (2.1) |  |  |
|  | TAPES-R General Adjustment | Women (114) | 3.4 (0.6) | -0.2 | [-0.5 ̶ 0.0] |
|  |  | Men (90) | 3.6 (0.5) |  |  |
|  | TAPES-R Social Adjustment | Women (114) | 2.9 (0.5) | **-0.2** | **[-0.4 ̶ -0.1]** |
|  |  | Men (90) | 3.1 (0.3) |  |  |
|  | TAPES-R Adjustment to Limitation | Women (114) | 2.4 (0.9) | -0.1 | [-0.3 ̶ 0.2] |
|  |  | Men (90) | 2.5 (0.8) |  |  |
| **B. Linear Regression Model Results** | | | | **Linear Regression** | |
| *Psychosocial Needs* | **Parameter** | **Sample Size (n)** | **Mean (Std)** | **Estimates** | **Confidence Intervals (95%)** |
|  | PROMIS Pain Interference | Women (122) | 56.8 (9.5) | 0.6 | [-1.7 ̶ 2.9] |
|  |  | Men (94) | 55.3 (8.9) |  |  |
|  | PROMIS Anxiety | Women (116) | 53.6 (10.1) | **2.9** | **[0.3 ̶ 5.6]** |
|  |  | Men (92) | 49.4 (10.3) |  |  |
|  | PROMIS Depression | Women (116) | 52.0 (10.8) | 1.9 | [-0.7 ̶ 4.4] |
|  |  | Men (92) | 48.7 (10.2) |  |  |
|  | PROMIS Emotional Support | Women (116) | 51.4 (9.1) | -0.2 | [-2.6 ̶ 2.2] |
|  |  | Men (91) | 53.4 (9.4) |  |  |

*Note:* Significant differences are bold.
